# Comparison of pregnancy rate between fresh embryo transfers and frozen-thawed embryo transfers following ICSI treatment 

**Published:** 2016-01

**Authors:** Zahra Basirat, Hajar Adib Rad, Sedigheh Esmailzadeh, Seyed Gholam Ali Jorsaraei, Karimollah Hajian- Tilaki, Hajar Pasha, Faeze Ghofrani

**Affiliations:** 1 *Infertility and Reproductive Health Research Center, Health Research Institute, Babol University of Medical Sciences, Babol, Iran.*; 2 *Department of Midwifery, Infertility and Reproductive Health Research Center, Health Research Institute, Babol University of Medical Sciences, Babol, Iran.*; 3 *Department of Social Medicine and Health, Babol University of Medical Sciences, Babol, Iran.*

**Keywords:** *Pregnancy rate*, *Intra cytoplasmic sperm injection*, *Cryopreservation*, *Embryo transfer*

## Abstract

**Background:**

The use of assisted reproductive technology (ART) is increasing in the world. The rate, efficacy and safety of ART are very different among countries. There is an increase in the use of intra cytoplasmic sperm injection (ICSI), single fresh embryo transfer (ET) and frozen-thawed embryo transfer (FET).

**Objective:**

The objective of this study was to compare pregnancy rate in fresh ET and FET.

**Materials and Methods:**

In this retrospective cross-sectional study 1014 ICSI-ET cycles (426 fresh ET and 588 FET) from 753 women undergoing ICSI treatment referred to Fatemezahra Infertility and Reproductive Health Research Center in Babol, Iran from 2008 to 2013 were reviewed.

**Results:**

There were no significant differences between biochemical pregnancy rate (23% versus 18.8%, OR 1.301; 95% CI .95-1.774), gestational sac (95.6% versus 100% in FET, OR 0.60; 95% CI 0.54-0.67), and fetal heart activity (87.2% versus 93.6% OR .46; 95% CI .16-1.32) in fresh ET and FET cycles, respectively. P< 0.05 was considered statistically significant for all measures.

**Conclusion:**

Although, the result showed no significantly difference between the fresh ET and the FET cycles, however the embryos are able to be stored for subsequent ART. Therefore, we recommend FET cycles as an option alongside the fresh ET.

## Introduction

Infertility prevalence, as one of the main problems in the society, is 15%, that may threaten the continuity of life (1). In Iranian population, primary and secondary infertility are 79% and 21% respectively (2). Frozen-thawed embryo transfer (FET) versus fresh embryo transfer (ET) is being performed mostly worldwide (3). Embryo cryopreservation can be considered to prevent ovarian hyperstimulaion syndrome (4). Some description is that may be that women who use cryopreservation have a better prognosis, with good ovarian reserve (5). Embryos can be cryopreserved at any stage, from zygote to blastocyst, and remain viable for at least several years (6).

In fresh cycles, the endometrium is artificially primed and the embryos could be cryopreserved and used in next cycles when the detrimental effects of high dose of hormones during controlled ovarian hyperstimulation (COH) are disappeared. Identically, in frozen-thawed embryo transfers, endometrial priming may be achieved with the use of estrogen and progesterone, and endometrial growth can be controlled more exactly in COH cycles than gonadotropins (7).

Embryo cryopreservation at the pronuclear, cleavage, and blastocyst stages has been allowed for multiple transfer cycles from single oocyte retrieval. As the transfer of cryopreserved embryos is less expensive than a second fresh cycle, fertility treatment costs can be optimized overally (4). Since embryos have been transferred successfully at any stage from zygote to blastocyst, ET is most generally done three days after oocyte pickup and fertilization (6). 

Besides, infertility is a common problem of couples of reproductive age and it is observed in one of five infertile couples FET has become an essential status of ART (8, 9). This method enables the means to diminish the number of transferred embryos, also, contributes to reducing the risk of multiple pregnancies (10, 11). 

Blastocyst transfers have led to an extension in implantation rates, higher pregnancy rates, and a reduction of high- order multiple gestations resulting from a decrease in the number of embryos transferred in comparison with embryos at the cleavage stage (12). 

It was shown that the single embryo transfer (SET) is a selection in FET, which can be used to decrease multiple pregnancy rates (13). 

There are two basic methods currently used for embryo cryopreservation: slow-freezing method and vitrification technique. Vitrification is thought to be better and more cost effective than slow-freezing. During the vitrification procedure with high condensation of cryoprotectants, minor intracellular ice crystal formation reduces cellular lesion and results in better fertility possible after thawing (14). 

According to some studies, vitrification may increase the embryo survival rate and decrease the rate of cooling damage (15, 16). In fresh ET, the uterine circumference after COH may also be less optimal for implantation (17).

The pregnancy rate in FET cycles is generally lower than that of fresh transferred embryos (18, 19). Newborns after FET have a better birth weight and fewer adverse perinatal outcomes than newborns after fresh ET (20–22). 

No extensions in the incidence of prematurity, low birth weight (LBW), neonatal death were established in the FET compared with the fresh ET (17). FET method provides a transfer of fewer embryos into the uterus and managing of addition embryos by cryopreservation for later use (23). The aim of this study was to compare the fertility success rate in fresh versus frozen embryo transfer.

## Materials and methods

**Study design and participants**

In this retrospective cross sectional study 1014 ICSI-ET cycles (426 fresh ET and 588 FET) from 753 women undergoing ICSI treatment referred to Fatemezahra Infertility and Reproductive Health Research Center in Babol, Iran from 2008-2013 were reviewed. 

Our inclusion criteria were: ICSI treatment using long protocol (GnRH agonist), endometrial thickness more than 8 mm, and having normal follicle stimulating hormone (FSH) of the third day of menstrual cycle. The women with natural cycles, having more than three cycles of ART, history of endocrine disorders (hypothyroidism and hyperthyroidism, diabetes, hyperprolactinemia), oocyte donation, Asherman's syndrome, history of surgical removal of endometriosis, ovarian cysts, leiomyoma, uterine septum, uterine anomalies in hysterosalpingography (HSG), and hysteroscopy and gamete donation were excluded.

The following outcome measures included: maternal and paternal age at the time of embryo transfer, duration and cause of infertility (female factor includes tubal, endometriosis, hypothalamic, ovarian, uterine, and cervical, male and unexplained factors), type of infertility, type of transfer (fresh or freez), thickness of the endometrium on the day of transfer and history of infertility treatment. 

The study protocol was approved by the Ethics Committee of Babol University of Medical Sciences, Babol, Iran. All subjects signed the written consent forms.

**Ovarian stimulation and oocyte pickup**

High dose (HD) contraceptive pill started on the third day of menstrual cycle. Ovulation induction protocol (long protocol) was initiated for the development of multiple follicles using gonadotropin- releasing hormone (GnRh) agonists (Suprefact by Aventis, Germany Company) in the middle of the luteal phase of the cycle (21 days period). Then, on the third day of the next menstrual cycle, gonadotropin injection (IBSA, MERCK, Switzerlad Company) was done. 

Follicular growth monitoring was done by the gynecologist using vaginal ultrasound (May lab 40 Esaote Italy) and, if it was necessary the number of gonadotropin was increased. Human chorionic gonadotropin (HCG) 10/000 IU (EXIR Iran Company) was injected when at least three follicles with a diameter of 18 mm were appeared. Endometrial thickness (ml) was measured by transvaginal ultrasound (TVS) oocyte pickup was performed 34-36 hours after the HCG injection in the operating room under general anesthesia.

**Embryo transfer process and reproductive success**

Estradiol valerate tablet (2 mg/day) was administered to prepare the endometrium. Progesterone was administered if the endometrial thickness was greater than 8 mm on 10-12 day of cycle in TVS.

Luteal phase was supported by daily administration of two vaginal progesterone suppositories (cyclogest 400 mg manufactured (Barnstaple- Actavis UK) for 2 weeks. Oocytes were fertilized in vitro and, other similar grade of embryos were cryopreserved by vitrification method. 

Freeze-thawed embryos and fresh embryos (on the third day after ICSI) were transferred by abdominal ultrasound guidance with a full bladder. Embryos grade A, B and A+B was transferred. A grade ncluded: Blastomeres are equal, no fragmentation, clear sytoplasmic aspect and without subzonal space. B grade ncluded: May be one blastomere is not equal, weak sytoplasmic aspect and without subzonal space, 5- 10% fragmentation, and subzonal space may be seen. A+B grade ncluded: The same as A and B. Pregnancy test was performed on day 16 after embryo transfer by ELISA β-HCG. 

Validation of a successful implantation was done by detecting an increased β-HCG concentration (>25U/ml) 16 days post embryo transfer, and was defined as positive biochemical pregnancy. 

Clinical pregnancy was determined by a presence of a gestational sac and fetal heart activity by transvaginal ultrasound at 6 weeks of pregnancy (2 weeks after testing positive β-HCG). If the pregnancy is continued, progesterone and estradiol were administered.

**Statistical analysis**

Statistical analysis was done using the SPSS (Statistical Package for the Social Sciences, version 18.0, SPSS Inc, Chicago, Illinois, USA). Chi- square test was applied to compare categorical variable. Differences among variables of two ET groups were analyzed using t- test. P<0.05 was considered statistically significant for all measures.

## Results

The study results confirmed that from 357 woman in the Fresh emberyo transfer group and 396 woman in the Frozen- thawed emberyo transfer group undergoing ICSI, the majority of they were housewives (83.6% and 84.9%, respectively), and their husbands were self- employed (51% and 53.9%, respectively). Educational level frequency in women and their husbands was high school graduate (40% and 38.1%, respectively). Analysis of these data there was no significant difference between the two groups. Patients’ characteristics in the two groups are listed in table I. There were no significant differences regarding patient’s characteristics between the groups who were excepted for a prior history of ICSI and the most common cause of infertility in both groups was male factor (Table I). Table II presents the cycle characteristics by type of transferred embryo. The average number of embryos transferred in fresh group was 2.42±1.02 and freezing group was 2.52±0.98, that there was no significant difference between the two groups. Biochemical pregnancy rate was 23% in fresh ET group versus 18.8% in FET group (OR 1.301; 95% CI .95-1.77). The results between the two groups showed no difference (Table III).

**Table I:** Patient’s characteristics by type of transferred embryo

**Patient’s characteristics**	**Fresh emberyo transfer group** **(n= 588)**	**Frozen- thawed embryo transfer group** **(n= 426)**	**p-value** †
Female age (years) *†	30.5 ±5.3	30.1 ± 5.1	0.173
Male age (years ) *†	34.3 ± 6.2	34.1 ± 6.1	0.706
Cause of infertility n (%) ††			
Male factor	382 (65)	279 (65)	0.862
Female factor	163 (27.75)	97 (22.76)	0.131
Unexplained	78 (13.3)	71 (16.7)	0.621
Type of infertility n (%) ††			
Primary	488 (83)	355 (83.3)	0.886
Secondary	100 (17)	71 (16.7)	
Duration of infertility(years) *†	6.1 ±4.4	6.1 ± 4.1	0.795
History of infertility treatment (%) ††			
ICSI	91 (15.5)	123 (28.9)	0.001
IVF	12 (2)	9 (2.1)	0.937
IUI	172 (29.3)	119 (27.9)	0.647
Other (ZIFT, GIFT)	11 (1.9)	7 (1.6)	0.787

ICSI: Intracytoplastic Sperm Injection.

IVF: In vitro Fertilization.

IUI: Intrauterine insemination.

ZIFT: Zygote intra-Fallopian Transfer.

GIFT: Gamet intra-Fallopian Transfer.

* Data are presented as mean±SD.

† The data were assessed using t- test.

†† The data were assessed using Chi-square test.

**Table II:** Cycle characteristics by type of transferred embryo

**Cycle characteristics**	**Fresh emberyo transfer group** **(n= 588)**	**Frozen- thawed emberyo transfer group** **(n= 426)**	**p-value** †
Assisted hatching n (%) †			
Yes	7 (1.2)	6 (1.4)	0.478
No	581 (98.8)	419 (98.4)	
Grade of embryos (mean±SD) ††			
A*	1.4 ± 1.2	1.3 ± 1.1	0.414
B**	0.6 ± 0.9	0.7 ± 1.1	0.333
A+B***	0.3 ± 0.7	0.4 ± 0.8	0.049

*: A Blastomeres are equal, no fragmentation, clear sytoplasmic aspect and without subzonal space.

**: **B** **May be one blastomere is not equal, weak sytoplasmic aspect and without subzonal space, 5-10% fragmentation, and subzonal space may seen.**

***: A+ B The same as A and B.

† The data were assessed using Chi-square test.

†† The data were assessed using t- test.

**Table III:** Fertility rate on the biochemical and clinical pregnancy tests with the type of transferred embryo

**Diagnosis tests**	**Fresh embryo transfer group** **(n= 588) n (%)**	**Frozen- thawed embryo transfer group** **(n= 426) n (%)**	**Odds ratio** **(95% CI)**	**p-value** †
Biochemical (B- HCG) *†				
Positive	136 (23.1)	80 (18.8)	1.30 (0.95-1.77)	0.188
Negative	448 (76.2)	341 (80)		
Gestational sac†				
Positive	130 (95.6)	80 (100)	0.60 (0.54-0.67)	0.051
Negative	6 (4.4)	0		
Fetal heart activity†				
Positive	109 (87.2)	73 (93.6)	0.46 (0.16-1.32)	0.146
Negative	16 (12.8)	5 (6.4)		

β-HCG: Beta-Human Chorionic Gonadotropin.

† The data were assessed using Chi-square test.

## Discussion

It was found that there was no significant difference in fertility success rate between the fresh and the frozen transfer groups. In another study, also, there was no notable difference in clinical pregnancy rates, or ongoing pregnancy rate between the fresh transfer group and the vitrified- thawed transfer group (24). Aflatoonian *et al* reported that biochemical pregnancy rate was 27% (54/200) in the FET group and 22.1% (122/500) in the fresh ET group and biochemical pregnancy rate was comparable between FET and fresh ET (25).

To attain a further fertility rate, the choice methods are important for selection the best fresh embryos for transfer. Thus, the residual embryos that survive the freezing and thawing methods supposedly have a reduced fortune of implantation. However, the implantation and fertility rates did not diminish in the frozen group compared with the fresh embryo transfer group in this study.

Zhu *et al* showed higher gestation and implantation rates in frozen blastocysts than in fresh transfer cycles. The clinical pregnancy rate of fresh and frozen blastocyst transfer groups were 36.4% and 55.1%, respectively (p< 0.05) and the implantation rate of the fresh and frozen group was 25.2% and 37.0% (p<0.05) (26). Kuc *et al* showed that the clinical pregnancy rates of the vitrification and slow-freezing groups of day 5 or day 6 blastocysts were notably different. The clinical pregnancy rates of the slow- freezing and vitrification groups were 25.9% and 50.4% (p<0.05), respectively (27).

Belva *et al* reported that pregnancy rates were significantly higher in the FET group than fresh ET group (17). Takeshima *et al* reported the pregnancy rate per ET cycle remained almost fixed over four years: nearly 24% for fresh IVF-ET cycles, 20% for fresh ICSI cycles, and 32% for frozen embryo transfer cycles. Pregnancy rate per retrieval diminished each year for fresh cycles (28). In another study, the implantation, ongoing and clinical pregnancy rates were significantly higher in FET group (15). Different implantation rates in two groups may reflect distinct endometrial receptivity and better symmetry between embryo and endometrial development in frozen embryo cycles.

To illustrate the similar results in the current study between the fresh and frozen embryo transfers, multiple factors may be considered. The progressive methods of vitrification conclude more survival and better possible development after thawing. Salumets *et al* established a report that there was no relationship between embryo quality and biochemical pregnancy rate before cryopreservation (29). 

Also, Aflatoonian *et al* found that according to morphological grading of embryos, biochemical pregnancy was similar in frozen and fresh groups in their study (27%, 22%) (25).

It has been reported that embryos that have better cleaved during the post- thaw time have the significantly higher fortune of implantation and a great number of uncleaved frozen embryos have chromosomal aberrations (30). 

Shapiro *et al* reported that large blastocyst diameter, early blastulation, and low preovulatory serum progesterone were better predictors of clinical pregnancy in fresh autologous cycles. They expressed that embryo- endometrium asynchrony was an important factor in cycle failure and nominated when all these three variables were suboptimal, the embryos should be freezed for later use under further optimal status (31). Endometrial receptivity and symmetry between the embryo and endometrium were very significant in cryopreserved- thawed and in fresh embryo transfer cycles. Cryopreserved embryos transferred in a natural ovulatory cycle resulted in preferable clinical outcome than stimulated cycles (14, 26, 32). 

The poisonous effects of heavy concentration of cryoprotectant factors in vitrification may affect negatively the embryos (33). The type criteria used in the choice of embryos for cryopreservation vary mainly between the different ART programs. Salumets *et al* confirmed the fundamental function of the embryo quality in the success of frozen embryo transfer. 

This study showed that better embryo morphology and faster blastomere cleavage rate were freely associated with improved delivery rate after frozen embryo transfer. They observed high delivery rates after frozen embryo transfers with moderate quality (grade 3A) embryos (14.9%) and embryos having two to three blastomeres (13.6%) (29).

Moreover, the current study carried out on cryopreservation of all embryos, only in patients who had enough number of good quality embryos suitable for freezing. This tactics were recommended only in the patients who have adequate embryos proper for cryopreservation. In the current study, age difference between the two groups was not significant. 

According to the study by Ashrafi *et al* and Aflatoonian *et al*, the women age did not affect fertility rate in fresh ET and FET protocols whereas, in other studies it was statistically significant (17, 25, 34-36). Also, in Basirat *et al.*’s study maternal age was a predictive factor of success rate in ICSI treatment cycles (37). 

In our study, duration of infertility did not have significant difference between the groups. In other studies, duration of infertility in both groups showed no significant differences (25, 15). 

Also, in our study, cause of infertility was not significant difference between the groups. Other studies have had similar results with our study (15, 24, 25, 38). Finally, the over plus embryos would be vitrified for frozen embryo transfer to recover the cumulative pregnancy rate. 

As long as the results are valid for vitrified- thawed cycles, all present blastocysts can be vitrified in patients for whom fresh blastocyst transfer is inappropriate, such as those with a history of repeated failed fresh embryo transfers, patients at risk of OHSS and those in need for preimplantation genomic diagnosis.

## Conclusion

Our results showed that there was no significant difference in fertility success rates between the fresh and the frozen embryo transfer groups. 

The ART methods are costly, need a notable requirement of time and energy for infertile couples. Therefore, detection of the affecting factor is an important method to correct previous failure embryo transfer and increase fertility. The other factors affecting fertility rate and their indication for ART outcome need to be better considered.
